# Large local reactions and systemic reactions to insect stings: Similarities and differences

**DOI:** 10.1371/journal.pone.0231747

**Published:** 2020-04-16

**Authors:** Patrik Tripolt, Lisa Arzt-Gradwohl, Urban Čerpes, Karin Laipold, Barbara Binder, Gunter Johannes Sturm

**Affiliations:** 1 Department of Dermatology and Venerology, Medical University of Graz, Graz, Austria; 2 Allergy Outpatient Clinic Reumannplatz, Vienna, Austria; Loyola University Health System, UNITED STATES

## Abstract

**Background:**

Large local reactions (LLR) to Hymenoptera stings were considered as IgE-mediated late-phase inflammatory reactions. However, in older studies, most patients with LLR were skin test positive, but only around 50% had detectable sIgE determined by the RAST system.

**Methods:**

Data of 620 patients were evaluated retrospectively: 310 patients who suffered from LLR and 310 patients with previous systemic sting reactions (SSR). We aimed to clarify if sIgE can generally be detected by the CAP system in patients with LLR; sIgE levels and clinical parameters were compared between patients with LLR and SSR.

**Results:**

Positive sIgE levels were detected in 80.7% of patients with LLR, and in 95.2% of patients with SSR (p<0.001). Of the 310 patients with LLR, 80.6% had a LLR with a size of 10-20cm, whereas 19.4% had swellings >20cm, with a mean duration of seven days. In only 2.9% of patients, LLRs occurred after stings on the trunk, while 14.8% of SSR resulted from stings on this site (p<0.001). Similarly, LLR were also less frequent on the capillitium compared to SSR (8.1% versus 26.2%; p = 0.035)

**Conclusions:**

LLR usually persisted over seven days and about one fifth of patients had swellings greater than 20cm. Contrary to SSR, LLR were less frequently observed on the capillitium and on the trunk. In most patients with LLR, sIgE could be detected. However, total IgE and sIgE levels to bee or vespid venom did not differ between patients with LLR and SSR.

## Introduction

In the general population, the prevalence of Hymenoptera stings ranges from 56.6% to 94.5%. [1] The main clinical presentations of Hymenoptera venom allergy are large local reactions (LLR) at the sting site and systemic sting reactions (SSR). A LLR has been defined as a swelling exceeding a diameter of 10 cm that lasts for more than 24 hours. [2] The prevalence of LLR ranges from 2.4% to 26.4%. [3] The involved mechanisms of large local reactions remain undefined; an IgE-dependent late-phase inflammatory reaction has been suggested. [4] The risk of a future SSR in subjects with LLR is generally considered low. [5, 6] In a recent study, 24% of patients with a previous LLR suffered from a SSR after being re-stung. [7] However, the quality of the study design was debated. [8] Another study reported that patients with a single LLR had a low risk for a future SSR while there was no risk of SSR in presence of at least two previous consecutive LLR. [9]

Mild SSR usually include flushing, urticaria, and angioedema. Dizziness, dyspnea, and nausea are typical examples of moderate reactions, while anaphylactic shock and loss of consciousness, or even cardiac or respiratory arrest, all define a severe SSR. [10] In European epidemiological studies the rate of reported SSR ranges from 0.3% to 7.5%. [3]

In older studies, most of patients with LLR were skin test positive, but only approximately half of the patients had detectable specific IgE (sIgE), when sIgE was determined by the RAST system. [5] Available studies were published decades ago and the methods used are mostly outdated. [4] Generally, there is no association between the concentration of venom-sIgE and the reactivity status of the individual patient. [2] Therefore, our aim was to investigate potential immunological differences between SSR and LLR. We wanted to clarify if sIgE can be detected by the ImmunoCAP system in patients with LLR. Furthermore, sIgE, sIgG_4_, and tryptase levels as well as other clinical parameters (e.g. sting site, presence of inhalant allergies, antihypertensive medication) were compared between patients with LLR and SSR.

## Methods

### Patients

For this retrospective study, data of 620 patients were evaluated. Three hundred and ten consecutive patients who suffered from a LLR and 310 consecutive patients with a history of a SSR who presented at the Department of Dermatology and Venereology of the Medical University of Graz between October 2013 and October 2018 were included in the study. When patients presented at the clinic, data concerning the sting reaction (e.g. sting site, symptoms, duration, culprit insect etc.) were recorded as well as the clinical history and concomitant medication. All data were obtained from a database where anonymized data concerning sting reactions are collected. By signing the informed consent form, patients agreed that their data and their stored blood samples could be used for further investigations in relation to insect venom allergy (ethical approval no. 25–465 ex 12/13; ethics committee of the Medical University of Graz).

### Serological tests

Specific and total IgE (tIgE) levels, tryptase levels as well as sIgG_4_ levels in the patients’ sera were measured using ImmunoCAP 1000 (Thermo Fisher Scientific, Waltham, USA). sIgE levels greater than 0.35kU/L were considered positive. Tryptase levels > 11.4μg/L were considered elevated.

### Skin tests

Intradermal tests (0.02ml of 0.01, 0.1 and 1μg/ml) with purified honeybee and vespid venom preparations (ALK-Abelló, Hørsholm, Denmark) were performed in all patients with SSR. Results were considered positive in the presence of a wheal greater than 5 mm in diameter and concomitant erythema. Skin prick testing (10, 100, and 300μg/ml) was done with purified honeybee and vespid venom preparations (ALK-Abelló) in all patients with LLR. Prick testing was positive in cases of a wheal larger than 3 mm in diameter at any concentration tested.

Intradermal tests were solely done in patients with SSR to keep the diagnostic procedure in patients with LLR minimally invasive. Prick tests were omitted in patients with SSR to expedite the diagnostic process. Therefore, only descriptive statistics are available.

### Statistical analyses

IBM SPSS Statistics 25.0 (IBM, Somers, USA) was used for statistical analyses. Categorical data were analyzed using the chi-square test. Data were tested for normality using the Kolmogorov-Smirnov test. As no data were normally distributed, the Mann-Whitney *U* test was used for between-group comparisons. The level of significance was set at 0.05.

## Results

### Clinical data

The patients’ ages ranged from 5 to 84 years, 108 (34.8%) patients with LLR were male and 202 (65.2%) female while 154 (49.7%) patients with SSR were male and 156 (50.3%) female (see Table 1).

**Table 1:** Demographic data.

	large local reaction	systemic sting reaction	*p*-value
*No*. *of patients*	310	310	
*age range (mean age) [years]*	5–84 (44)	16–80 (47)	p = 0.008
*sex*			
male	108 (34.8%)	154 (49.7%)	p<0.001
female	202 (65.2%)	156 (50.3%)	
*Inhalant allergy*			
no	196 (63.2%)	240 (77.4%)	p<0.001
yes	114 (36.8%)	70 (22.6%)	
*antihypertensive treatment*			
no medication	256 (82.6%)	259 (83.5%)	p = 0.316
betablocker	13 (4.2%)	13 (4.2%)	
ACE-inhibitor	7 (2.3%)	14 (4.5%)	
ATII-antagonist	12 (3.9%)	13 (4.2%)	
ACE-inhibitor + betablocker	5 (1.6%)	7 (2.3%)	
ATII-antagonist + betablocker	11 (3.6%)	4 (1.3%)	
*tryptase level*			
≤ 11.4μg/L	53 (17.1%)	284 (91.6%)	p = 0.096
> 11.4μg/L	0 (0.0%)	20 (6.5%)	
*sting localization*			
trunk	9 (2.9%)	46 (14.8%)	p<0.001
upper extremities	121 (39.0%)	116 (37.4%)	
lower extremities	98 (31.6%)	75 (24.2%)	
head	37 (11.9%)	61 (19.7%)	
• face and lips	34 (91.9%)	45 (73.8%)	p = 0.035
• capillitium	3 (8.1%)	16 (26.2%)	

Of the 310 patients with LLR, 250 (80.6%) patients reported a LLR with a size of 10–20 cm, whereas 60 (19.4%) had swellings greater than 20 cm. Patients with SSR predominantly suffered from moderate to severe sting reactions: 17 (5.5%) had a grade I, 203 (65.5%) a grade II, 88 (28.4%) a grade III and 2 (0.6%) a grade IV reaction according to the classification of Ring and Messmer. [11]

Large local reactions occurred in only 2.9% of patients after stings on the trunk, while 14.8% of SSR resulted from stings on this site (odds ratio (OR): 5.192; 95% confidence interval (CI): 2.489–10.831; p<0.001). Similarly, LLR were also less frequent on the capillitium compared to SSR (8.1% versus 26.2%; OR: 4.030; CI: 1.086–14.951; p = 0.035; see Table 1).

Eighty-three patients with LLR gave information about the duration of the large local reaction occurred. The median duration of the swellings was 7 days (range 1–21 days). At least one previous LLR in the past was reported by 143 patients (46.1%).

Interestingly, inhalant allergies were observed more frequently in patients with LLR compared to patients with SSR (36.8 and 22.6% respectively; OR: 0.501; CI: 0.353–0.713; p<0.001). Risk factors for severe SSR are elevated tryptase levels and possibly the intake of antihypertensive drugs. However, neither tryptase levels (p = 0.645), nor the intake of antihypertensive drugs (p = 0.831) (see Table 1) differed between patients with LLR and SSR.

### Determination of specific IgE and IgG_4_

Venom-sIgE was detected in 80.7% of patients with LLR and 95.2% of patients with SSR (p<0.001). In detail, 16.8% of patients with LLR had positive sIgE levels to bee venom, 31.0% to vespid venom and 32.9% to both venoms. The results for patients with SSR differed: 6.8% had positive levels to bee venom, 38.1% to vespid venom and 50.3% to both venoms (see Table 2).

**Table 2:** Sensitization to insect venoms. sIgE values greater than 0.35kU/L were considered positive.

	large local reaction	systemic sting reaction	*p*-value
No. of patients	310	310	
bee venom	52 (16.8%)	21 (6.8%)	p<0.001
vespid venom	96 (31.0%)	118 (38.1%)	
both venoms	102 (32.9%)	156 (50.3%)	
negative: <0.35kU/L	59 (19.0%)	15 (4.8%)	
no data	1 (0.3%)		

Assuming values >0.1 kU/L as positive in patients with low total IgE (<50 kU/L), sIgE was detectable in 86.4% of patients with LLR and in 99.4% of patients with SSR (p<0.001). However, mean sIgE levels to bee or vespid venom (p = 0.988, p = 0.757, respectively) and tIgE levels (p = 0.386) did not differ between patients with LLR and SSR. Additionally, we determined sIgG_4_ levels in each 50 patients with LLR and SSR. Mean sIgG_4_ levels to bee venom did not differ between patients with LLR and SSR (0.08mg/L vs. 0.12 mg/L, respectively, p = 0.958) but mean sIgG_4_ levels to vespid venom were significantly higher in patients with SSR (0.12 mg/L (LLR) vs 0.88 mg/L (SSR); p = 0.001).

### Skin testing

Intradermal tests were performed in 303 patients with SSR. 20 (6.6%) were mono-sensitized to bee venom, 158 (52.1%) to vespid venom, 110 (36.3%) were double-sensitized, and 15 (5.0%) were negative to both, bee and vespid venom.

Only 110 (35.5%) patients with LLR had a positive prick test. In detail, 29 (26.4%) patients were sensitized to bee venom, 73 (66.4%) to vespid venom, and 8 (7.3%) were sensitized to both venoms.

## Discussion

In our study, serum sIgE could be detected in up to 86.4% of the patients with LLR using the ImmunoCAP system. This is contrary to previous studies where sIgE to insect venoms has been detected in only approximately 50% of adults and 70% of children with LLR. [5, 6, 12–14] This can be explained by the lower sensitivity of the old radioactivity-based RAST system used in all previously published studies. A higher sensitivity and a higher frequency of double sensitization to bee and vespid venom in patients with SSR were observed after the introduction of the enzyme-based CAP system. [15, 16] We could confirm both, the higher sensitivity and more double positive results with the CAP compared to the intradermal tests in patients with SSR.

In our study cohort, specific IgE levels did not differ between patients with LLR and SSR. This was consistent with our previous findings that sIgE determination was not able to distinguish between asymptomatic sensitization, LLR, and SSR. [17] Furthermore, baseline sIgE levels were not suitable to differentiate between untreated patients and patients who received venom immunotherapy. [18] Consequently, other regulatory factors such as sIgG_4_ may play a role or, in case of LLR, another pathomechanism must be considered. There is usually a correlation between sIgE and sIgG_4_ –the higher sIgG_4_ the lower sIgE, particularly after venom immunotherapy. In patients with LLR and sensitization to vespid venom, sIgG_4_ was significantly higher compared to patients with SSR on the group level. However, no appropriate threshold could be determined to differentiate between LLR and SSR. This is in agreement with previous studies. [13, 14]

Graft et al. reported that 84% of patients with LLR demonstrated venom-specific IgE antibodies as measured by intradermal tests with concentrations up to 1.0 μg/ml, suggesting that sIgE may be involved in the pathomechanism of LLR. In our study, only 36.3% were positive to bee and vespid venom in the skin prick test. It is well known that the sensitivity of the prick test is lower compared to the intradermal test in patients with SSR. [2] This may be the same in patients with LLR; however, only about one third had positive prick tests. One may speculate that higher venom doses are needed to diagnose LLR: using a maximum dose of 0.1 μg/ml in intradermal testing identified only 43% of patients with LLR. [12]

In patients with SSR, the intradermal test and the sIgE determination performed almost equally with negative tests in 5.0% and 4.8% of patients, respectively.

Compared to SSR, LLR were less frequently seen on the trunk and capillitium, which is probably due to the thicker reticular dermis, which makes LLR less likely. Compartmentalization could be another reason why LLR occur more frequently on the extremities and face. Usually, the treatment of LLR is symptomatic and relies on the use of oral antihistamines and topical corticosteroids. When edema is spreading, systemic corticosteroids should be added for a few days. [19] If patients with LLR should carry an adrenaline auto-injector is controversially discussed in the literature [20, 21] but there is a tendency to not prescribing epinephrine to patients with LLR. [13, 22] Subcutaneous venom immunotherapy (VIT) has been shown to reduce the size and duration of LLR. [23] Therefore, VIT could be considered a treatment option in patients with recurrent and troublesome LLRs. [10] Patients may refrain from taking emergency medication when stung on the trunk and capillitium as the risk for LLR is low.

In beekeepers, the percentage of atopic patients was significantly higher among those showing anaphylactic symptoms than in those without anaphylaxis. [24] Conversely, Settipane et al reported that the frequency of atopy was not higher in patients with anaphylaxis. However, asthmatics had significantly more severe reactions when anaphylaxis occurred. [25] Taken together, data on atopy and anaphylaxis are scarce and contradictory. Previous studies showed that the number of patients with atopy was similar among patients who suffered from LLRs or SSRs. [19, 26] In contrast to that, tIgE levels (as one marker for atopy) did not differ between the two study groups but inhalant allergies were more frequently seen in patients with LLR than in those with SSR in our study cohort; this was also reported by another study. [27] LLR are probably IgE-dependent late-phase inflammatory reactions [4]. In allergic rhinitis, cross‐linking of IgE–FcεRI complexes on dendritic cells, mast cells and basophils activate these cells to release of inflammatory mediators causing classic allergic reactions. Depending on patient susceptibility, allergic individuals may develop a late‐phase nasal allergic response orchestrated by Th2 cytokines such as IL‐5, IL‐9 and IL‐13. [28] It could be speculated that an individual predisposition to these late-phase reactions may be the cross link between inhalant allergies and LLR.

Elevated tryptase levels [29, 30] and possibly antihypertensive treatment [30] indicate a higher risk for severe SSR. Therefore, we looked for possible differences between patients with LLR and SSR. However, tryptase levels and the intake of antihypertensive drugs were similar in both groups.

To sum up, median duration of LLR was 7 days and about one fifth suffered from swellings greater 20 cm. Using present methods, we were able to show that sIgE can be detected in 86.4% of patients with LLR indicating an important role of sIgE in the development of LLR. However, since sIgE levels did not differ between patients with LLR or SSR, sIgE determination is still not a suitable tool to distinguish between these reactions and cannot be used as predictive parameter in terms of the outcome of future sting reactions.

## Supporting information

S1 Data (XLSX)
